# Insect‐resistant soybean genotypes accumulate rutin and its *O*‐methylated derivative narcissin, a more potent flavonol targeting *Anticarsia gemmatalis* digestive and detoxification enzymes and reducing larval survival

**DOI:** 10.1002/jsfa.70640

**Published:** 2026-04-18

**Authors:** Jessica Nunes de Assis, Valquira Joana Medina Pinheiro, Jodieh Oliveira Santana Varejão, Ian de Paula Alves Pinto, André Luiz Lourenção, Nathália Silva Oliveira, Giovanna Cassia Mendes Moraes, Eduardo Vinícius Vieira Varejão, Maria Goreti de Almeida Oliveira, Humberto Josué de Oliveira Ramos

**Affiliations:** ^1^ Laboratory of Enzymology and Biochemistry of Proteins and Peptides, Department of Biochemistry and Molecular Biology Universidade Federal de Viçosa, UFV, BIOAGRO/INCT‐IPP Viçosa Brazil; ^2^ Departamento de Química Universidade Federal de Viçosa Viçosa Brazil; ^3^ Escola Superior de Agricultura “Luiz de Queiroz” – USP Piracicaba Brazil; ^4^ Núcleo de Análise de Biomoléculas, NuBioMol Universidade Federal de Viçosa Viçosa Brazil

**Keywords:** insect pest, LC‐MS, methylated flavonol, molecular docking, NMR

## Abstract

**BACKGROUND:**

Soybean underpins Brazil's agricultural, yet its sustainability is threatened by lepidopteran pests such as *Anticarsia gemmatalis*. Although insect‐resistant cultivars have long been associated with the constitutive accumulation of quercetin‐derived flavonols, the biochemical mechanisms underlying this resistance remain poorly understood. In particular, the role of flavonol *O*‐methylation in enhancing plant defense has not been demonstrated.

**RESULTS:**

Here we combined metabolite identification, biological assays, and structure‐based modeling to investigate flavonol‐mediated resistance in soybean. The resistant genotype IAC 17 was found to accumulate not only rutin (quercetin 3‐*O*‐rutinoside; [M + H]^+^ = 611 Da) but also its *O*‐methylated derivative isorhamnetin 3‐*O*‐rutinoside (narcissin; [M + H]^+^ = 625 Da). The 625 Da compound was purified by reversed‐phase high‐performance liquid chromatography and structurally confirmed by liquid chromatography–tandem mass spectrometry and nuclear magnetic resonance analyses. Chemical methylation of rutin using methyl iodide generated methylated derivatives that were evaluated in dietary bioassays with *A. gemmatalis* larvae. Both rutin and its methylated derivatives reduced survival, but *O*‐methylation markedly enhanced toxicity: methylated rutin caused approximately 95% mortality within 5 days, whereas non‐methylated rutin required about 17 days to reach comparable lethality. Molecular docking against the 70 most abundant larval midgut proteins revealed strong binding of both flavonols to proteases, cytochrome P450 enzymes, glutathione *S*‐transferases, and membrane‐associated proteins.

**CONCLUSION:**

These findings support a dual‐ligand model in which rutin and narcissin cooperatively disrupt digestive and detoxification pathways in the insect midgut. The discovery of a constitutive flavonol *O*‐methylation branch identifies narcissin as a potent defensive metabolite and provides mechanistic targets for breeding and metabolic engineering strategies aimed at developing soybean cultivars with improved resistance. © 2026 The Author(s). *Journal of the Science of Food and Agriculture* published by John Wiley & Sons Ltd on behalf of Society of Chemical Industry.

## INTRODUCTION

Soybean (*Glycine max* (L.) Merrill) is Brazil's leading agricultural commodity and a cornerstone of global food and feed systems, supplying the most widely used plant protein for animal production and the world's second most consumed vegetable oil.^1^ Yet its sustainability is continually challenged by lepidopteran pests such as *Anticarsia gemmatalis* (velvetbean caterpillar). Management in Brazil still relies largely on the use of chemical and biological insecticides, including insect growth regulators, microbial products such as *Bacillus thuringiensis* and baculoviruses, and newer compounds such as spinosyns and diamides. Although these compounds are generally more selective than older insecticide classes, their intensive use may still impose environmental costs through contamination of soils and water bodies.

Breeding programs have developed soybean cultivars with resistance to insect pests, including genotypes such as IAC 17, IAC 100, and IAC 24, which exhibit distinct spectra and levels of resistance to soybean insects.^2^, ^3^ These cultivars express resistance mechanisms such as antibiosis and antixenosis against both chewing insects (defoliating caterpillars) and sucking pests (e.g., stink bugs and aphids). However, the genetic and biochemical determinants of resistance remain incompletely resolved.^4^

Phenotypic and biochemical studies point to quercetin‐derived flavonols, especially rutin (quercetin 3‐*O*‐rutinoside), as candidate resistance metabolites.^5^, ^6^, ^7^, ^8^ However, rutin alone does not fully explain resistance breadth across genotypes, suggesting that additional pathway branches or co‐accumulating metabolites contribute to the phenotype.

Recent metabolomic work in resistant backgrounds revealed high levels of a methylated flavonol structurally analogous to rutin and putatively assigned as isorhamnetin 3‐*O*‐rutinoside (narcissin), with related precursors (isorhamnetin and isorhamnetin 3‐*O*‐glycoside) also detected in IAC 100 and IAC 24.^7^, ^8^

These observations raise the hypothesis that resistant genotypes harbor a constitutive *O*‐methylation branch of quercetin metabolism, potentially shaping both metabolite stability/transport and target engagement in the insect midgut. Thus, our study had two aims: (i) to purify and structurally confirm the *m*/*z* 625 compound from IAC 17 leaves as isorhamnetin 3‐*O*‐rutinoside using reversed‐phase liquid chromatography (RP‐HPLC), liquid chromatography–tandem mass spectrometry (LC‐MS/MS), and nuclear magnetic resonance (NMR); and (ii) to evaluate functional relevance by comparing molecular docking of rutin and narcissin across the 70 most abundant midgut proteins of *A. gemmatalis* prioritized from RNA‐Seq.^9^ By integrating chemical identification with structure‐based modeling, we test whether *O*‐methylation complements rutin's activity through ligand‐specific, yet overlapping binding to digestive proteases, cytochromes P450, glutathione‐*S*‐transferases (GSTs), and membrane/structural proteins. The results provide mechanistic context for resistance and nominate *O*‐methyltransferases, glycosyltransferases, and their regulators as candidates for genetic dissection and marker development,^10^ offering actionable guidance for breeding strategies that leverage both rutin and narcissin as a dual‐flavonol defense module.

## MATERIALS AND METHODS

### Plant growth

Seeds of the insect‐resistant soybean genotype IAC 17 were obtained from the Agronomic Institute of Campinas (IAC) and germinated in a greenhouse at the Federal University of Viçosa (UFV). Plants were cultivated in pots containing 2.0 kg of a soil–sand–substrate mixture (3:1:1) and maintained for 30 days under controlled conditions of 25 ± 3 °C and 70 ± 10% relative humidity. Four pots were used, each containing three seeds. Leaf samples were collected at the V5 developmental stage,^11^ immediately frozen in liquid nitrogen, and stored at −80 °C. A total of 66.55 g of fresh material was obtained. The leaves were then oven‐dried at 40 °C, ground, and pulverized with an industrial blender, yielding 18.23 g of powdered material.

### Flavonoid extraction

Flavonoids were extracted from powdered leaf tissue following Hamad.^12^ Extraction was performed using a Soxhlet apparatus with 250 mL of 80% ethanol until exhaustion. Extracts were filtered and concentrated under vacuum to ~10 mL, diluted with 25 mL distilled water, and partitioned with petroleum ether (3 × 50 mL) and chloroform (3 × 50 mL). The aqueous phase (25 mL) was collected and kept at 4 °C for 72 h. An aliquot of 300 μL was analyzed by LC‐MS (UHPLC‐QqQ, Agilent Technologies, Santa Clara, CA, USA), and the remaining 24.7 mL was dried using a SpeedVac (Thermo Fisher Scientific, Waltham, MA, USA. The final dried residue weighed 2.20 g.

### Flavonoid separation by preparative HPLC

Preparative HPLC (LC‐20AR, UV–visible SPD‐10AV, Waters Corporation, Milford, MA, USA) was used to separate flavonoids from the IAC 17 extract. An aliquot of 200 μL containing 20 mg of extract was injected using a 200 μL loop. The mobile phase consisted of water with 0.1% formic acid (i) and acetonitrile with 0.1% formic acid (ii). A linear gradient was applied at 1 mL min^−1^ over 60 min. Subfractions were monitored at 375 nm, manually collected, and analyzed by MS. Fractions corresponding to retention times of 36–37, 38, 39, and 40 min were collected.

### Flavonoid analysis by LC‐MS/MS

For quantification, 150 mg of powdered tissue was extracted with 800 μL methanol–isopropanol–acetic acid (20:79:1, v/v/v), as described by Gómez *et al*.^7^ Aliquots of 5 μL were analyzed using an ultra‐high‐performance liquid chromatography system coupled to a triple‐quadrupole mass spectrometer (QqQ 6420, Agilent) in positive mode with multiple reaction monitoring (MRM). Transitions included rutin (*m*/*z* 611 > 303 for quantification; *m*/*z* 611 > 145 for confirmation) and isorhamnetin‐3‐*O*‐rutinoside (*m*/*z* 625 > 317 for quantification; *m*/*z* 625 > 303 for confirmation). Data were processed with Skyline, and peak areas were converted to μg g^−1^ fresh tissue using calibration curves.

Fragmentation analyses (MS^2^/MS^3^) were performed using a 2D ion trap mass spectrometer (Amazon, Bruker Daltonics, Billerica, MA, USA). Samples (5 μL) were injected into a UPLC nanoAcquity system (Waters) coupled to a ProteCol C18 GHQ303 column (3 μm, 300 μm × 150 mm; Trajan Scientific and Medical, Ringwood, VIC, Australia) at 0.6 μL min^−1^. Gradient elution used 0.1% formic acid in water (A) and 0.1% formic acid in acetonitrile (B) with the following program: 2–50% B (20 min), 50–98% B (10 min), 98% B (20 min), 98–2% B (2 min), and re‐equilibration at 2% B (8 min). Data acquisition was performed in positive mode with MRM and MS^3^ scans using HyStar 3.2, and spectra were processed with DataAnalysis 4.0 (Bruker Daltonics).

### NMR

An aliquot of 20 mg dried extract was dissolved in 700 μL deuterated methanol (CD_3_OD) and analyzed by ^1^H‐NMR using a Bruker Ascend 400 MHz spectrometer.

### Structural modeling with ColabFold

Amino acid sequences of candidate proteins were modeled using ColabFold (v1.5.5), installed locally on a Linux workstation equipped with an NVIDIA RTX GPU. The colabfold_batch pipeline was executed with Amber relaxation (‐amber), GPU‐based relaxation (‐use‐gpu‐relax), and the AlphaFold2_ptm model (‐model‐type alphafold2_ptm). Parameters included a single recycle iteration (‐num‐recycle 1) and relaxation constraints (‐relax‐stiffness 10,‐relax‐max‐iterations 10 000). Final models were ranked by predicted Local Distance Difference Test (pLDDT) scores, and top‐ranked conformations were used for docking.

### Molecular docking with AutoDock Vina

Molecular docking was performed using AutoDock Vina (v1.2.5). Ligand structures of rutin and isorhamnetin‐3‐rutinoside were retrieved from PubChem and prepared using AutoDockTools (PDBQT format, Gasteiger charges, torsional flexibility).

Protein sequences corresponding to the 70 most highly expressed midgut proteins of the velvetbean caterpillar (*A. gemmatalis*), identified from RNA‐Seq data,^9^ were retrieved from the respective dataset and used as targets for structural prediction. The resulting models generated with ColabFold were prepared for docking by adding polar hydrogens and removing water molecules. Docking grids were centered on predicted active or binding sites, with dimensions encompassing the entire binding pocket. Each docking run generated at least 20 poses, and the top‐ranked conformation based on binding affinity (kcal mol^−1^) was selected for interaction analysis.

### Analysis of protein–ligand interactions and chemical group proximities

Docking complexes were analyzed using custom Python scripts implemented with the Biopython (https://biopython.org/) and MDAnalysis (https://www.mdanalysis.org/) libraries.^12^, ^13^, ^14^

Distances between ligand chemical groups (hydroxyl, methoxy, rhamnose, and rutinose moieties) and protein residues were calculated, applying a cutoff of 4.0 Å to define interactions. Amino acids were classified into chemical categories (hydrophobic, polar, charged, aromatic), and frequencies of contacts were recorded. Interaction data were summarized as proximity matrices, reporting the number of contacts per group and residue category. These matrices enabled comparative assessment of rutin and narcissin binding modes across different protein targets.

### Methylation reaction of rutin with methyl iodide and potassium carbonate

The methylation of rutin was carried out according to Kozłowska *et al*.,^15^ with minor modifications. In a Pyrex glass tube, 20 mg rutin (molecular mass = 610.52 g mol^−1^, purity = 94%, 0.031 mmol) was suspended in 3 mL anhydrous solvent (acetone, acetonitrile/methanol 2:1 v/v, or acetone/methanol 2:1 v/v). To this suspension, 0.0342 g (0.248 mmol, 8 equiv.) anhydrous potassium carbonate and 19.2 μL (0.31 mmol, 10 equiv.) methyl iodide (molecular mass = 141.94 g mol^−1^, density = 2.28 g mL^−1^, 99%) were added. The resulting mixture was maintained at 56 °C for 60 h under magnetic stirring.

After completion of the reaction, 2 mL ethanol was added, and the mixture was filtered to remove residual potassium carbonate. The retained solids were washed with ethanol, and the combined filtrates were concentrated to dryness under reduced pressure using a rotary evaporator. The crude products were obtained in yields of 23.4, 66.3, and 73.7 mg when acetone, acetonitrile/methanol, and acetone/methanol were used as the reaction solvents, respectively.

The methylated derivatives of rutin were analyzed by HPLC coupled to triple‐quadrupole MS/MS (Agilent Technologies) operated in MRM mode.

Methylation progress and the number of methyl substituents were monitored using the following MRM transitions: *m*/*z* 611 > 303 (unmethylated rutin), *m*/*z* 625 > 317 (monomethylated), *m*/*z* 639 > 331 (dimethylated), *m*/*z* 653 > 345 (trimethylated), and *m*/*z* 667 > 359 (tetramethylated).

Prior to LC‐MS/MS analysis and caterpillar survival assays, the methylated products were desalted and purified using reversed‐phase solid‐phase extraction cartridges with C18 sorbent.

### Survival assay of *A. gemmatalis* larvae fed on diets supplemented with rutin and its methylated derivatives

Eggs of *A. gemmatalis* were obtained from the National Soybean Research Center (CNP‐Soja, EMBRAPA, Londrina, PR, Brazil) and maintained at the Insect Rearing Laboratory of the Department of Biochemistry and Molecular Biology, Federal University of Viçosa (UFV). Insects were reared under controlled environmental conditions: a temperature of 25 ± 5 °C, relative humidity of 70 ± 10%, and a photoperiod of 14 h light:10 h dark.

Adult moths were kept in 50 × 50 cm cages lined internally with A4 paper sheets to facilitate oviposition. Each cage was supplied with a nutritive solution absorbed into cotton and placed on a Petri dish at the bottom of the cage. Oviposition began approximately 3 days after adult emergence, with eggs deposited on the inner paper lining. The paper sheets containing egg masses were cut into strips (2.5 × 10 cm) and transferred to 500 mL plastic containers with lids perforated by a circular opening (~2 cm diameter) covered with nylon mesh for aeration. These containers were placed in a climatic chamber maintained at 25 °C, 60 ± 10% relative humidity, and a 14 h light photoperiod controlled by a timer connected to daylight‐type fluorescent lamps. These rearing conditions ensured proper egg development and larval hatching.

Newly hatched larvae (first instar) were initially maintained on the artificial diet without any treatment or supplementation. Bioassays were initiated with second‐instar larvae, which were individually transferred to the artificial diet according to the method described by Hoffmann‐Campo *et al*.^6^ Purified rutin and its methylated derivatives were incorporated into the artificial diet (60 g total) at a final concentration equivalent to 0.641% rutin (6.41 g kg^−1^), based on the natural content reported in resistant soybean genotypes.^6^

Each treatment was conducted in two independent replicates using 32 larvae per treatment. Larvae were individually maintained in plastic trays containing isolated compartments (cages) measuring 4.0 × 4.0 × 5.0 cm (width × length × height). The bioassays were performed under controlled environmental conditions at 25 °C, 60% relative humidity, and a 14 h photoperiod. The artificial diet corresponding to each treatment was provided individually and replaced every 24–48 h, or earlier when necessary, depending on larval consumption. Feeding and survival were monitored daily over a 20‐day period to record any mortality effects associated with each compound.

Larval survival was analyzed using the Kaplan–Meier method, and survival curves were compared among treatments using the log‐rank (Mantel–Cox) test, with statistical significance set at *P* < 0.05. Statistical analyses were performed in R version v4.3 using the survival package.

The experimental design included the following treatments: (i) negative control 1: diet + water; (ii) negative control 2: diet + reaction medium without rutin; (iii) positive control: diet + rutin (0.64%, 6.4 g kg^−1^); and (iv) methylation product treatment: diet + methylated rutin derivatives (0.64%, 6.4 g kg^−1^). This experimental approach was employed to assess the potential effects of both native and methylated rutin derivatives on the survival of *A. gemmatalis* larvae.

## RESULTS

### Extraction and purification of the flavonoid isorhamnetin 3‐*O*‐rutinoside from soybean leaves

**Figure 1:**
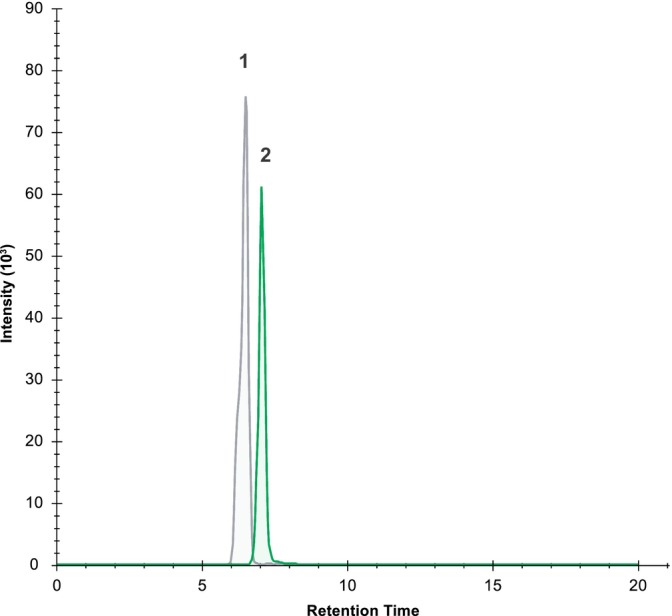
LC‐MS analysis of total metabolic extract from soybean leaves of genotype IAC 17. Extracted ion chromatograms were used to quantify the concentration of rutin and isorhamnetin 3‐*O*‐rutinoside using the computational package Skyline. Peak 1 corresponds to rutin (*t*_R_ 8.4 min, *m*/*z* 611 > *m*/*z* 303) and peak 2 (*t*_R_ = 8.9 min, *m*/*z* 625 > *m*/*z* 317) to isorhamnetin 3‐*O*‐rutinoside.

Low‐molecular‐weight metabolites commonly associated with soybean defense against chewing insects include flavonoids. Rutin (quercetin 3‐*O*‐rutinoside) is recognized for its significant role in plant defense against lepidopterans, and it was identified in leaf extracts of the IAC 17 and IAC 100 insect‐resistant genotypes. In addition to rutin, another glycosylated flavonol, the ion [M + H]^+^ at *m*/*z* 625, was putatively identified as isorhamnetin 3‐*O*‐rutinoside and found in high concentrations in the leaves of IAC 17 (Fig. 1). Thus, the first stage of this work was to confirm the structure of the ion [M + H]^+^ at *m*/*z* 625 by LC‐MS (MS^*n*^) and NMR.

Analysis of the MRM spectrum and chromatograms obtained from the IAC 17 extracts enabled detection of the protonated molecular ion peak [M + H]^+^ at *m*/*z* 611 for rutin, with a retention time (RT) of 8.3 min, and the molecular ion peak [M + H]^+^ at *m*/*z* 625 corresponding to the putative isorhamnetin 3‐*O*‐rutinoside, with an RT of 8.8 min (Fig. 1). Additionally, extracted ion chromatograms (EICs) were used to quantify the concentration of rutin and isorhamnetin 3‐*O*‐rutinoside in the IAC 17 leaves. The concentrations found were 13.7 μg g^−1^for rutin and 10.6 μg g^−1^ for the putative isorhamnetin 3‐*O*‐rutinoside.

For the separation of rutin and isorhamnetin 3‐*O*‐rutinoside in soybean IAC 17 leaves (Fig. 1), aliquots containing 200 μL (20 μg of extract) of the aqueous extract were injected into a preparative HPLC instrument for fractionation. The eluted fractions were monitored at 380 nm for flavonoid detection (Supporting Information, Fig. S1).

**Figure 2:**
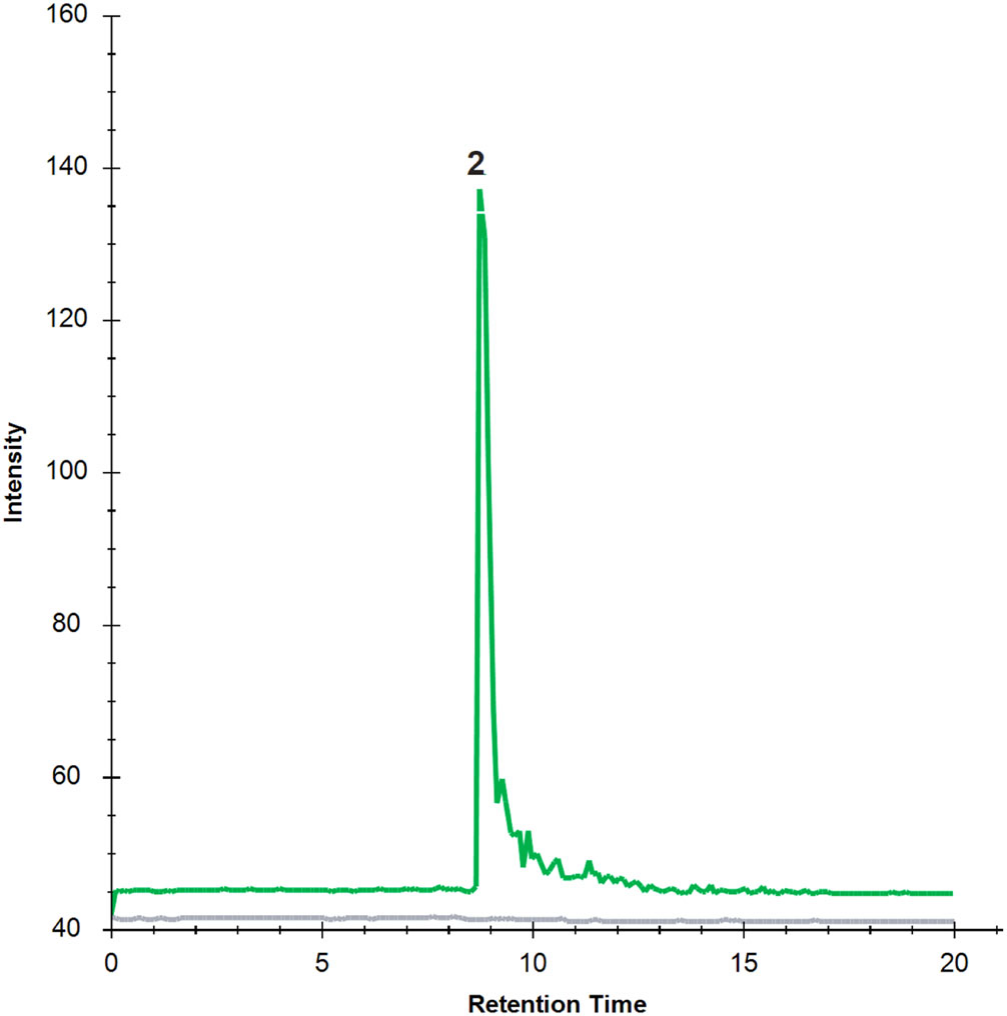
LC‐MS QqQ analysis of the 40.5 min retention time subfraction (Supporting Information, Fig. S1). Peak 2 corresponds to multiple reaction monitoring of isorhamnetin 3‐*O*‐rutinoside (extracted ion chromatograms for transition *m*/*z* 625 > *m*/*z* 317). No chromatographic signals were detected for rutin extracted ion chromatograms for transition *m/z* 611 > *m*/*z* 303).

**Figure 3:**
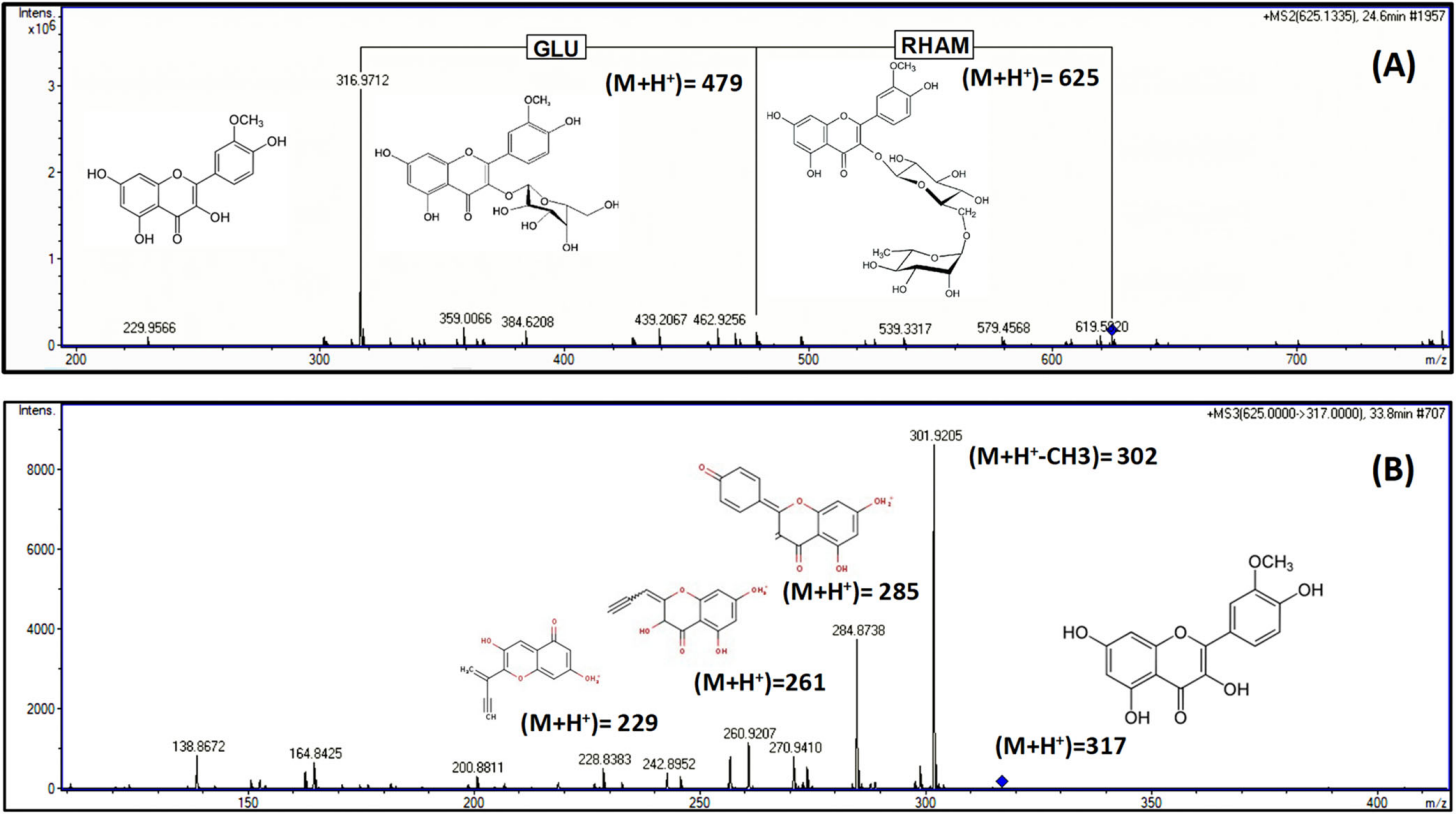
Fragmentation pattern analysis of ion *m*/*z* 625 by LC‐MS/MS^*n*^ using ion trap. (A) MS^2^ fragmentation spectrum of molecular ion *m*/*z* 625. Neutral losses for glucose (GLU, 162 Da) and rhamnose (RHAM, 146 Da) are indicated. (B) MS^3^ fragmentation spectrum for fragment *m*/*z* 317 was generated in (A) by the fragmentation of ion *m*/*z* 625. Blue diamonds indicate the molecular ion that was isolated for fragmentation. The indicated fragments were predicted using the CFM‐ID platform (https://cfmid.wishartlab.com/).

The presence of rutin in high concentrations has been observed in leaves of soybean genotypes resistant to pest insects. Using an untargeted LC‐MS approach, Gómez *et al*.^16^ detected the presence of an ion with *m*/*z* 625 in high concentrations in the IAC 17 genotype resistant to pest insects. Searches against NIST fragmentation libraries indicated a fragmentation spectrum showing high similarity to isorhamnetin 3‐*O*‐rutinoside_7_, marking the first detection of this flavonoid in soybean leaves. However, the molecular structure was not confirmed. To confirm the structure and methylation position, purified and enriched fractions for isorhamnetin 3‐*O*‐rutinoside (RT = 40.5; Fig. 2) were analyzed by LC‐MS/MS^*n*^ (Fig. 3).

Sequential fragmentation analyses were conducted to obtain detailed structural information. Fragmentation of the precursor ion *m*/*z* 625 resulted in the fragment ion *m*/*z* 317 [M − 146–162 + H]^+^, corresponding to losses of a glucose molecule (GLU, 162 Da) and a rhamnose molecule (RHAM, 146 Da). Fragmentation of glycosylated flavonoids by electrospray ionization–collision‐activated dissociation generates fragments more frequently at the *O*‐glycosidic linkage of the sugar to the aglycone ring. Therefore, it generates a fragment‐ion showing high intensity containing the aglycone (Fig. 3(A)). Thus, little structural information about the rings can be obtained from MS^2^ fragmentation. The structure of the methylated quercetin (isorhamnetin) was confirmed by isolation and fragmentation of the *m*/*z* 317 ion (Fig. 3(B)). The structures for some fragment‐ions were predicted using the CFM‐ID platform (https://cfmid.wishartlab.com/), indicating a fragment corresponding to a loss of the methyl group (M + H ^+^ −15 = 302). The structures from the fragment‐ions 285, 261, and 229 were also confirmed (Fig. 3(B)).

### Structural analysis by NMR

As seen earlier, after separation by HPLC, the major presence of isorhamnetin 3‐*O*‐rutinoside was detected in the fraction collected at 40 min. Therefore, this fraction was subjected to ^1^H‐NMR spectroscopy (400 MHz), using deuterated methanol (CD_3_OD) as solvent.

In the ^1^H‐NMR spectrum (Supporting Information, Fig. S2), two different signals were highlighted at *δ* 3.95 (a) and *δ* 3.97 (b), attributed to the hydrogen atoms of the methoxy (—OCH_3_) group located at position 3′ of the compound. The signal at *δ* 4.53 was also observed, corresponding to hydrogen H‐1″ in rhamnose, and the signal at *δ* 5.24 for hydrogen H‐1″ in glucose. Furthermore, the signals observed at *δ* 6.22 and *δ* 6.42 are related to hydrogens H‐6 and H‐8 of the aglycone. Lastly, the chemical shifts found for H‐5′, H‐6′, and H‐2′ were *δ* 6.91, *δ* 7.63, and *δ* 7.95, respectively. These data coincide with the chemical shift signals reported in the literature^17^, ^18^, ^19^ for isorhamnetin 3‐*O*‐rutinoside in the ^1^H‐NMR spectrum.

As mentioned above, two signals with different chemical shifts were observed at *δ* 3.95 and *δ* 3.97, corresponding to the hydrogen atoms in CH—O of the methoxy at position 3′ of the compound. Therefore, the presence of an isomer of isorhamnetin 3‐*O*‐rutinoside was suspected. For further information about the isomer, another signal with a chemical shift at *δ* 5.49 for hydrogen H‐1″ in glucose and the signal at *δ* 8.02 for hydrogen H‐2′ in the aglycone was detected (Table 1). Additionally, signals with chemical shifts at *δ* 6.04 and *δ* 6.08 were observed; however, due to the presence of some impurities in the sample, the data obtained from ^1^H‐NMR and ^13^C‐NMR were considered inconclusive for the characterization of the isomer.

**Table 1:** Chemical shifts of ^1^H (400 MHz, CD_3_OD) assigned to the flavonoid isorhamnetin 3‐*O*‐rutinoside and the detected isomer

Position	Isorhamnetin 3‐*O*‐rutinoside experimental (MeOD)	Isorhamnetin 3‐*O*‐rutinoside – Sang *et al*.^17^ (MeOD)	Isomer experimental (MeOD)	Isorhamnetin 3‐*O*‐rubinobioside – Gallegos‐Olea *et al*.^20^ (DMSO)
	δH (ppm)	δH (ppm)	δH (ppm)	δH (ppm)
6	6.22	6.20		6.25
8	6.42	6.41		6.48
5′	6.91	6.92		6.96
6′	7.63	7.61		7.57
2′	7.95	7.95	8.02	8.04
1″	5.24	5.25	5.49	5.49
1‴	4.53	4.54		4.48
3′‐OCH_3_ a	3.95	3.96	3.95	3.91
3′‐OCH_3_ b	3.97	3.96	3.97	

In summary, analysis of the ^1^H‐NMR spectrum also revealed the presence of a compound with a structure highly similar to isorhamnetin 3‐*O*‐rutinoside. Notably, the signals mainly correspond to hydrogens in the glycosidic unit at H‐1″ and in the aglycone at H‐2′, which corroborates its structural similarity (Table 1). According to the literature, it was observed that the compound isorhamnetin 3‐*O*‐robinobioside is a flavonoid that is an isomer of Isorhamnetin 3‐*O*‐rutinoside. After analyzing the ^1^H‐NMR data, it was concluded that, besides the glycosidic unit of rhamnose, there is also another glycosidic unit in this compound corresponding to galactose. There was also confirmation of a methoxy group (—O—CH_3_) at position 3′ due to a signal at *δ* 3.91, similar to isorhamnetin 3‐*O*‐rutinoside.^20^

### Rutin and narcissin show distinct affinities across detoxification and digestive enzymes

To explore potential molecular targets in the insect midgut, molecular docking analyses were performed using predicted structures of the 70 most highly expressed midgut proteins of *A. gemmatalis* based on RNA‐Seq data.^9^ Two flavonols were evaluated: rutin and its methylated derivative narcissin (isorhamnetin‐3‐rutinoside).

Thus, we first modeled the proteins of the velvetbean caterpillar (*A. gemmatalis*)^9^ and performed molecular docking against two flavonols: rutin and its methylated derivative narcissin (isorhamnetin‐3‐rutinoside). Predicted complexes with negative Vina binding energies (indicative of favorable interactions) were retained and are listed in the Table 2, which reports the best scores for each ligand per target.

**Table 2:** Docking scores (binding energies, kcal mol^−1^) of rutin and isorhamnetin‐3‐*O*‐rutinoside (narcissin) against predicted midgut proteins of *Anticarsia gemmatalis*

Activity_inferred	TRINITY_KEY	Score_Rutin	Score_Narcissin
Cytochrome P450 CYP6B53	TRINITY_DN4705_c0_g1_i4	−9504	−8449
Sodium/calcium exchanger regulatory protein 1	TRINITY_DN159_c0_g1_i4	−8332	−8438
Trypsin, alkaline C‐like	TRINITY_DN1737_c0_g1_i1	−8622	−8275
Serine protease (fragment)	TRINITY_DN548_c0_g1_i4	−7831	−7728
Serine protease 2	TRINITY_DN548_c0_g1_i1	−7767	−7706
Trypsin, alkaline B‐like	TRINITY_DN1202_c0_g1_i3	−7232	−7461
Serine protease 1 (fragment)	TRINITY_DN8474_c0_g1_i2	−7387	−7253
Unknown	TRINITY_DN7322_c0_g2_i1	−7212	−6917
Trypsinogen	TRINITY_DN1937_c0_g1_i1	−6442	−6676
Uncharacterized protein	TRINITY_DN840_c0_g3_i1	−6284	−6652
SFRICE_024614	TRINITY_DN2674_c0_g1_i7	−6897	−6529
Insect intestinal mucin	TRINITY_DN2843_c0_g1_i1	−6506	−6497
Uncharacterized protein LOC113400367	TRINITY_DN2772_c0_g1_i5	−5621	−6251
Ribosomal protein L10	TRINITY_DN8424_c0_g1_i5	−6246	−6214
Collagenase	TRINITY_DN548_c1_g1_i6	−5706	−6039
Peptidase S1 domain‐containing protein	TRINITY_DN193_c2_g1_i12	−6424	−5722
KH domain‐containing protein 3‐like	TRINITY_DN797_c0_g1_i3	−4883	−5345
KH domain‐containing protein 3‐like	TRINITY_DN797_c0_g1_i4	−5737	−5339
Calphotin‐like	TRINITY_DN797_c0_g1_i2	−5393	−5202
V‐type proton ATPase proteolipid subunit	TRINITY_DN5933_c1_g1_i1	−3614	−5.09
Serine protease 2	TRINITY_DN4088_c0_g1_i5	−6,08	−5043
KH domain‐containing protein 3‐like	TRINITY_DN797_c0_g1_i6	−5127	−4897
Calphotin‐like	TRINITY_DN797_c0_g1_i7	−5076	−4.82
TRASH domain‐containing protein (fragment)	TRINITY_DN17361_c0_g1_i1	−4628	−4755
KH domain‐containing protein 3‐like	TRINITY_DN623_c0_g1_i5	−5266	−4751
SFRICE_004753	TRINITY_DN3649_c0_g1_i3	−2165	−3.27
Glutathione‐*S*‐transferase epsilon class	TRINITY_DN4963_c0_g2_i1	−3858	−3094
Trypsin, alkaline B‐like isoform X2	TRINITY_DN756_c3_g2_i1	2044	−1707
SFRICE_024614	TRINITY_DN2674_c0_g1_i5	0.952	−1181
Trypsin 3 (fragment)	TRINITY_DN2233_c0_g2_i1	−3923	0.563

Docking analyses revealed moderate to strong predicted binding affinities (approximately −5 to −9.5 kcal mol^−1^) for both flavonols across several digestive and detoxification‐related proteins, including serine proteases, trypsins, cytochrome P450 enzymes, GSTs, and membrane‐associated proteins (Table 2). Rutin showed slightly stronger predicted affinities for several proteases and GSTs, whereas narcissin displayed comparable or slightly improved interactions with some membrane‐associated targets.

Some isoform‐specific differences were observed among protease targets, suggesting that subtle structural variations may influence ligand preference (Table 2). Thus, methylation of rutin (narcissin) slightly reshaped the binding landscape‐gaining affinity on some membrane/transport targets, but marginally weakened it on several proteases – yet both ligands converged on gut proteases and xenobiotic‐processing enzymes as plausible targets. The full set of per‐target scores enabling these comparisons is provided in the table.

### Protease and P450 targets: convergent evidence for *O*‐methylation–driven repositioning

**Figure 4:**
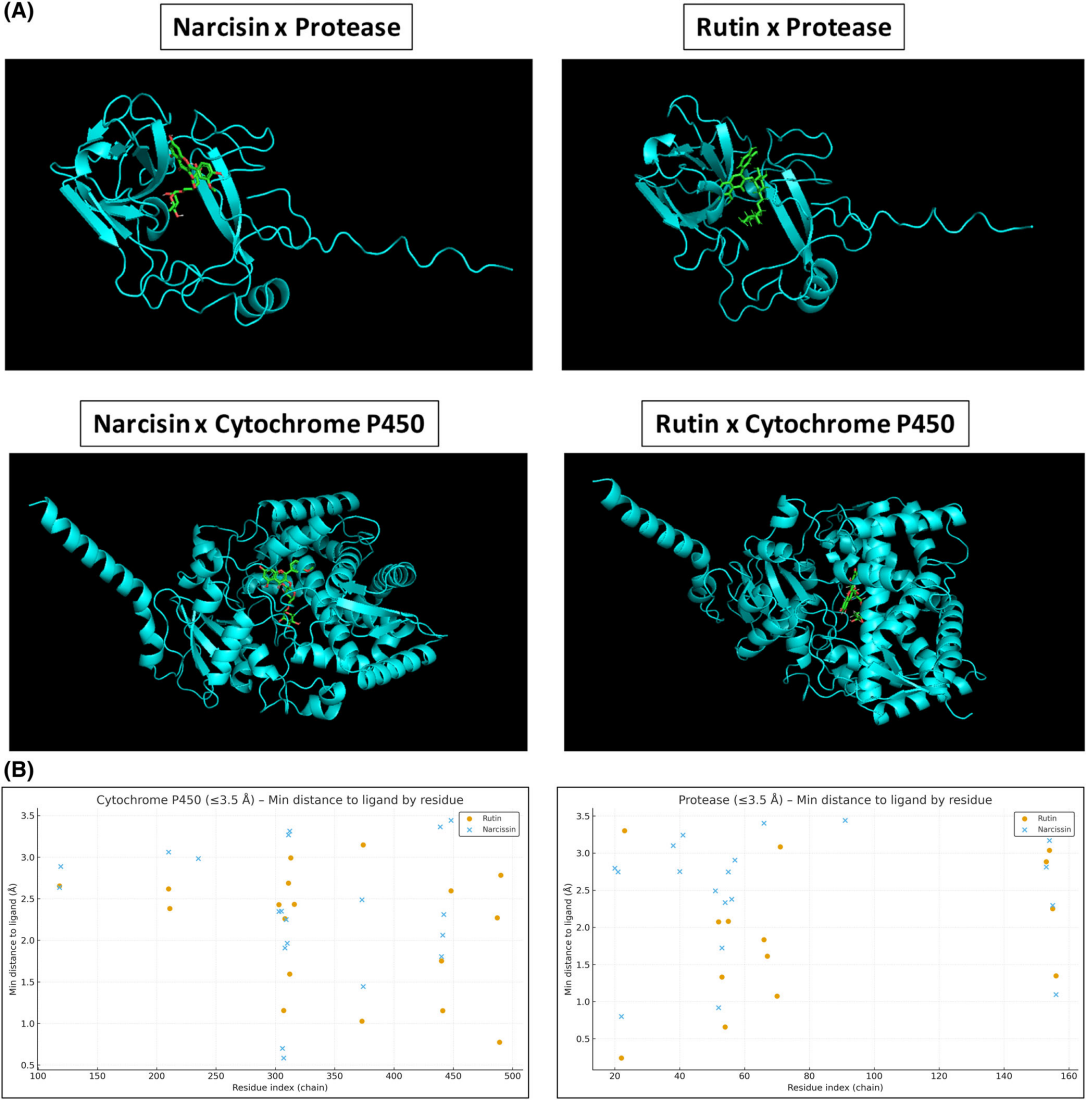
(A) Molecular docking of rutin and isorhamnetin‐3‐*O*‐rutinoside (narcissin) with two key *Anticarsia gemmatalis* midgut proteins: a trypsin‐like protease (TRINITY_DN1737_c0_g1_i1) and cytochrome P450 CYP6B53 (TRINITY_DN4705_c0_g1_i4). Ligands are shown in stick representation within the predicted binding pockets. (B) Residue‐level contact analysis of the complexes shown in (A), displaying the minimum distance between each residue and the ligand. Profiles highlight distinct interaction patterns of rutin *versus* narcissin with protease and P450 active sites.

To further examine ligand–protein interactions, the top‐ranked docking poses for a trypsin‐like protease and a cytochrome P450 were analyzed by mapping residues within 4.0 Å of the ligands (Fig. 4; Supporting Information, Tables S1 and S2). Both flavonols interacted with partially overlapping sets of residues, although narcissin generally showed a shift toward more hydrophobic pockets enriched in Leu and Val residues. In contrast, rutin maintained additional contacts consistent with its phenolic hydroxyl groups. These differences suggest that *O*‐methylation slightly modifies the positioning of the flavonol within the binding pockets of digestive and detoxification enzymes.

### LC‐MS/MS (triple‐quadrupole multiple reaction monitoring) analysis of rutin methylation products

**Figure 5:**
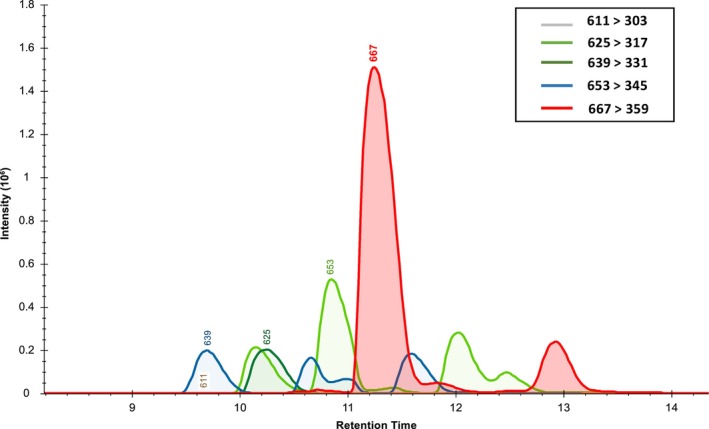
LC‐MS/MS (QqQ‐MRM) analysis of methylated rutin derivatives obtained from the reaction of rutin with methyl iodide. Extracted ion chromatograms corresponding to the transitions *m*/*z* 611 → 303 (unmethylated rutin), *m*/*z* 625 → 317 (monomethylated rutin), *m*/*z* 639 → 331 (dimethylated rutin), *m*/*z* 653 → 345 (trimethylated rutin), and *m*/*z* 667 → 359 (tetramethylated rutin). The chromatographic profile demonstrates the progressive methylation of rutin, with the appearance of successive peaks representing the stepwise addition of methyl groups. Acetone as the reaction solvent favored the formation of higher methylated derivatives, consistent with the reaction yields obtained after purification.

The methylation of rutin with methyl iodide in the presence of acetone as solvent was monitored by liquid chromatography coupled to triple‐quadrupole mass spectrometry (LC‐MS/MS, QqQ) operated in multiple reaction monitoring (MRM) mode (Fig. 5). The extracted ion chromatograms revealed distinct peaks corresponding to rutin and its successive methylated derivatives. The parent compound, unmethylated rutin, was detected at the transition *m*/*z* 611 → 303, while the methylated products were identified at *m*/*z* 625 → 317 (monomethylated), *m*/*z* 639 → 331 (dimethylated), *m*/*z* 653 → 345 (trimethylated), and *m*/*z* 667 → 359 (tetramethylated).

The chromatographic profile showed a gradual increase in the relative abundance of methylated species, confirming the progression of the methylation reaction and the formation of multiple substitution products under the applied conditions. These data indicate that acetone favored the generation of higher methylated derivatives, consistent with the reaction yields obtained after purification.

### Survival and development of *A. gemmatalis* larvae fed on diets supplemented with methylated rutin derivatives

**Figure 6:**
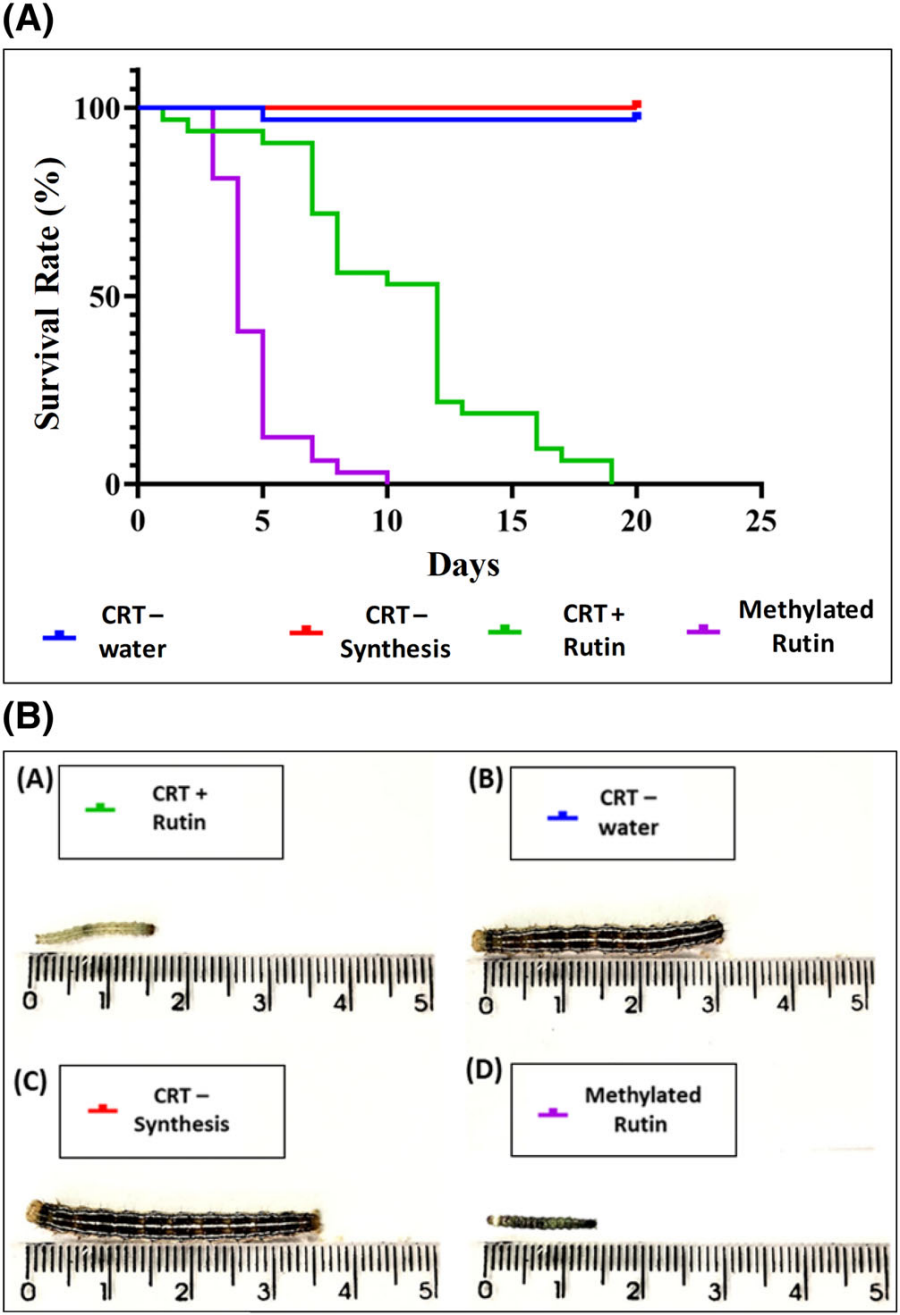
Survival and development of *Anticarsia gemmatalis* larvae fed artificial diets supplemented with rutin and its methylated derivatives. (A) Kaplan–Meier survival curves showing the effects of different dietary treatments over 20 days. (B) Representative images of larvae after 10 days of feeding under each treatments and controls (CRT): (A) rutin control (CRT + Rutin: diet + rutin 0.64%); (B) water control (CRT ‐ Water: diet + deionized water); (C) reaction medium control (CRT ‐ Synthesis: diet + synthesis reagents); and (D) methylation product treatment (Methylated Rutin: diet + methylated rutin derivatives 0.64%, 6.4 g kg^−1^). Larvae exposed to rutin or its methylated derivatives showed reduced size and delayed development compared with both negative controls.

The biological effects of rutin and its methylated derivatives on *A. gemmatalis* larvae were evaluated through survival assays and developmental monitoring (Fig. 6). Kaplan–Meier survival curves revealed a marked reduction in larval survival for both rutin and its methylated derivatives compared with the negative controls.

Survival data were analyzed using Kaplan–Meier curves and compared using the log‐rank test (Supporting Information, Table S3). A significant overall difference was detected among treatments (*χ* ^2^ = 190.67, df = 3, *P* = 4.38e−41). No difference was observed between the water and synthesis controls (*P* = 0.3173). In contrast, the methylated rutin treatment caused the fastest mortality, with a median survival time of ~5 days, whereas the rutin positive control showed a median survival time of ~12 days. In the Cox model restricted to estimable groups, the methylated rutin treatment increased the hazard of death by 6.60‐fold relative to the positive control (95% CI = 3.33–13.06; *P* = 6.32e‐08). Larvae fed diets containing methylated rutin derivatives showed a rapid decline in survival, with an LT_50_ of 4.0 days (95% CI: 3.6–4.5), whereas larvae fed rutin exhibited a slower mortality response with an LT_50_ of 12.0 days (95% CI: 10.8–13.2).

Thus, the rate and extent of mortality differed significantly between treatments. Larvae fed diets containing methylated rutin derivatives (0.64%) exhibited a drastic reduction in survival, reaching approximately 95% mortality after only 5 days of exposure. In contrast, larvae fed with non‐methylated rutin (0.64%, 6.4 g kg^−1^) showed a slower decline in survival, reaching comparable mortality levels only after about 17 days – a delay of nearly 12 days relative to the methylated treatment. These results demonstrate that methylation substantially enhanced the insecticidal potency of rutin under the tested conditions.

Figure 6(B) presents representative images illustrating the developmental differences among larvae after 10 days of feeding under the different treatments. Although no statistical analysis was performed to quantitatively assess larval growth, the images provide a qualitative comparison of developmental trends. Larvae in the rutin control (Fig. 6(A)) and methylated rutin treatment (Fig. 6(D)) appeared to exhibit delayed growth and reduced body size compared with those in the water control (Fig. 6(B)) and reaction medium control (Fig. 6(C)). Thus, these observations support the general trend that both rutin and its methylated derivatives negatively affected larval development.

Supporting Information, Fig. S3, shows the mean pupal weight of *A. gemmatalis* after 20 days of larval development under control conditions. No significant differences were observed between the treatments, indicating that the reaction medium alone did not affect larval development or pupal biomass. However, this result may also be influenced by the reduced number of individuals reaching the pupal stage due to the high mortality observed during larval development.

### Tissue‐specific accumulation of rutin and narcissin in soybean genotypes

**Figure 7:**
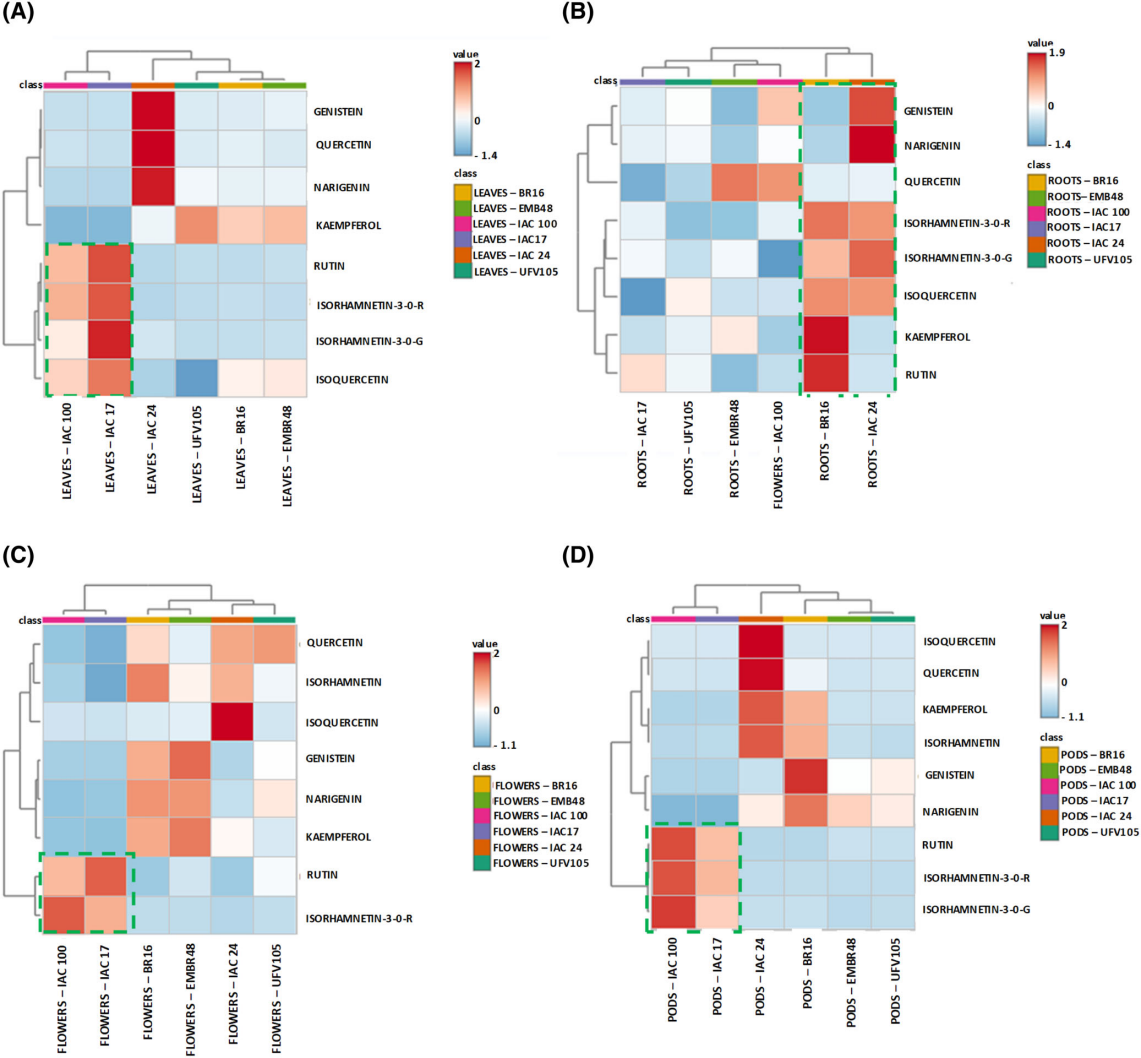
Tissue‐specific accumulation patterns of flavonoids in soybean genotypes revealed by LC‐MS/MS (QqQ) analysis. Heatmap clustering of selected flavonoid compounds analyzed by targeted LC‐MS/MS in multiple reaction monitoring mode: (A) leaves; (B) roots; (C) flowers; and (D) pods. The analysis included key flavonoids and intermediates of the isoflavonoid and flavonol glycosylation/methylation pathways, such as naringenin, genistein, kaempferol, and quercetin, as well as glycosylated and methylated quercetin derivatives: rutin, isorhamnetin, isorhamnetin glucoside (isorhamnetin‐3‐*O*‐glucoside), isoquercitrin, and isorhamnetin rutinoside (isorhamnetin‐3‐*O*‐rutinoside, narcissin). Resistant genotypes (IAC 17 and IAC 100) showed elevated levels of rutin and narcissin particularly in leaves, flowers, and pods, indicating systemic activation of the flavonol *O*‐methylation pathway in tissues most exposed to insect feeding.

Targeted LC‐MS/MS (QqQ) profiling of flavonoids across soybean tissues revealed marked differences between resistant (IAC 17 and IAC 100) and susceptible genotypes (Fig. 7). Heatmap clustering analyses based on normalized ion intensities demonstrated that both rutin and its *O*‐methylated analogue isorhamnetin‐3‐*O*‐rutinoside (narcissin) accumulated at substantially higher levels in the resistant genotypes, particularly in leaves, flowers, and pods. In contrast, root tissues exhibited comparatively low concentrations of both compounds across all genotypes.

These results indicate that the flavonol *O*‐methylation pathway is not restricted to foliar tissues but is also active in reproductive organs of resistant soybean plants. The high accumulation of rutin and narcissin in leaves and pods – key feeding and oviposition sites for *A. gemmatalis* – suggests that constitutive activation of this metabolic branch contributes to systemic chemical defense and may play a central role in the enhanced insect resistance observed in IAC 17 and IAC 100 genotypes.

## DISCUSSION

Despite decades of research and the availability of resistant soybean germplasm, the genetic determinants of resistance to insect pests remain only partially resolved. Rutin (quercetin 3‐*O*‐rutinoside) has long been implicated in antifeedant activity, yet its contribution alone does not fully explain the breadth or durability of resistance across genotypes.^6^ Our results showed that resistant plants accumulate, alongside rutin, the *O*‐methylated flavonol isorhamnetin 3‐*O*‐rutinoside (narcissin) and that both compounds are present at biologically relevant levels. This chemical evidence, together with structure‐based analyses indicating complementary binding to gut proteases and detoxification enzymes, suggests that resistance is not a single‐metabolite trait but a metabolic module in which methylation and glycosylation tailor flavonol engagement with insect midgut targets.

In this work, we unequivocally confirmed that a constitutive flavonoid *O*‐methylation branch in resistant genotypes provides a concrete and testable entry point for the genetic characterization of the regulatory pathway. It nominates clear candidate loci, including *O*‐methyltransferases, glycosyltransferases, transporters, and upstream transcriptional regulators, as targets for quantitative trait loci/genome‐wide association studies (QTL/GWAS) mapping, expression QTL/methylation QTL integration, and marker development. Because docking links this chemistry to plausible protein targets in the insect gut, these metabolic and genetic markers can be prioritized by predicted functional impact rather than abundance alone. In practical terms, the *O*‐methylation module can serve as a trait for marker‐assisted selection and a framework for mechanistic studies (enzyme assays, gene editing) that connect pathway genetics to field‐level resistance.

Our LC‐MS and NMR analyses provide structural confirmation that resistant soybean genotypes accumulate the *O*‐methylated quercetin glycoside isorhamnetin‐3‐*O*‐rutinoside (narcissin) alongside rutin. These results extend previous untargeted observations of an *m*/*z* 625 feature in resistant soybean backgrounds^7^, ^8^ by confirming its identity and relative abundance. Notably, IAC 17 accumulates narcissin at levels comparable to rutin, suggesting constitutive flux through quercetin *O*‐methylation and glycosylation pathways in this resistant background.

To explore potential molecular targets, we docked rutin and narcissin against the 70 most highly expressed midgut proteins of *A. gemmatalis*. Both ligands showed predicted binding to digestive proteases, cytochrome P450 enzymes, GSTs, and membrane‐associated proteins. Although several proteases tended to favor rutin, some membrane‐associated targets and specific trypsin isoforms showed slightly stronger interactions with narcissin, suggesting partially complementary binding profiles across gut targets.

Structural contact analyses of representative complexes (a trypsin‐like protease and a cytochrome P450) showed that narcissin tends to occupy deeper hydrophobic pockets enriched in Leu and Val residues, whereas rutin maintains additional polar and aromatic interactions consistent with its phenolic hydroxyl groups. These differences provide a structural explanation for the modest affinity shifts observed in docking analyses. Thus, *O*‐methylation consistently repositions the flavonol to favor hydrophobic packing, while the unmethylated rutin maintains polar/aromatic engagement, an interplay that rationalizes the small but recurrent score differences we observe.

These biophysical tendencies dovetail with broader pharmacokinetic/biochemical expectations: *O*‐methylation of flavonoids increases metabolic stability, reduces susceptibility to conjugative clearance, and improves membrane transport, often translating into higher cellular exposure.^21^, ^22^, ^23^, ^24^ In a plant–insect context, this can mean that narcissin persists longer and accesses deeper/hydrophobic regions of midgut enzymes or membranes, while rutin (though less apolar) can form stronger polar/aromatic interactions and cover complementary targets such as certain protease loops or polar portals on P450s/GSTs. Given that resistant soybean genotypes (e.g., IAC 17, IAC 100, IAC 24) accumulate both compounds,^8^ our data support a dual‐ligand defense hypothesis: co‐occurring rutin and narcissin jointly inhibit digestive and detoxification machinery, reducing larval performance and survival on resistant plants.

Finally, we note limitations and next steps. Docking narrows hypotheses but does not quantify kinetics or permeability under midgut conditions (alkaline pH, protease‐rich milieu, peritrophic matrix). We therefore propose: (i) enzyme inhibition assays with purified trypsins and cytochromes P450 (including the isoforms highlighted here); (ii) thermal‐shift or surface plasmon resonance assays to validate binding; (iii) protease activity measurements in midgut extracts supplemented with purified rutin/narcissin and mixtures to test additivity/synergy; and (iv) dietary bioassays on *A. gemmatalis* larvae using fractions enriched for each flavonol and defined ratios.

Chemical methylation of rutin with methyl iodide generated a series of methylated derivatives detected by LC‐MS/MS (QqQ, MRM), including mono‐, di‐, tri‐, and tetramethylated forms. The chromatographic profiles confirmed progressive methylation under the reaction conditions, producing compounds structurally analogous to isorhamnetin‐3‐*O*‐rutinoside. Importantly, these methylated forms exhibited distinct biological behavior compared to rutin, suggesting that methyl substitution substantially modifies the bioactivity of the flavonol scaffold.

Bioassays with *A. gemmatalis* larvae demonstrated that both rutin and its methylated derivatives significantly reduced larval survival. Notably, methylated rutin induced rapid mortality, whereas non‐methylated rutin produced a slower toxic effect, indicating that methylation substantially enhances the biological activity of the flavonol scaffold. These findings reinforce that the *O*‐methylation pathway of flavonols, already detected in resistant soybean genotypes IAC 17 and IAC 100,^8^ represents a critical biochemical adaptation enhancing the efficacy of defense metabolites. The production of narcissin (isorhamnetin‐3‐rutinoside) appears to confer a more potent and rapid protective effect than rutin itself, underscoring the potential importance of methylated flavonoids as key determinants of insect resistance in soybean.

Tissue‐specific profiling revealed higher accumulation of rutin and narcissin in leaves, flowers, and pods of resistant genotypes (IAC 17 and IAC 100), whereas roots showed comparatively low levels. This spatial distribution suggests that flavonol *O*‐methylation contributes to constitutive chemical defense in tissues most exposed to herbivory. This systemic deployment of rutin and narcissin likely contributes to durable field‐level resistance and highlights the adaptive value of metabolic specialization in soybean–insect interactions.

In a broader mechanistic framework, our data support the view that flavonol methylation is not merely a secondary chemical decoration, but part of a defense‐oriented reprogramming of the soybean phenylpropanoid pathway.^25^, ^26^ Herbivory is known to trigger large‐scale transcriptional shifts from growth‐related metabolism toward defense metabolism, including activation of flavonoid biosynthesis and associated signaling cascades. Recent soybean studies further reinforce this interpretation: chewing‐related cues increased the expression of defense‐associated genes such as PR1, PR10/Bet v‐1, and gmFLS1, consistent with a shift toward flavonol accumulation,^8^, ^27^, ^28^ while genetic disruption of a soybean UDP‐glycosyltransferase (GmUGT) enhanced resistance to leaf‐chewing insects and altered both flavonoid profiles and expression of flavonoid‐biosynthetic genes. In parallel, our own metabolic evidence indicates that resistant genotypes constitutively accumulate both rutin and narcissin, which is consistent with sustained flux through FLS‐, UGT‐, and *O*‐methyltransferase‐dependent branches of the pathway. Because *O*‐methylation generally increases flavonoid hydrophobicity, membrane affinity, and metabolic stability, the conversion of rutin‐derived scaffolds into methylated analogues such as narcissin may enhance persistence and target engagement in the insect gut, thereby strengthening the defensive value of this constitutive flavonol module.

These findings also fit well within the evolutionary arms race model of plant–herbivore interactions, in which plants diversify defensive metabolites while insects evolve countermeasures centered on the midgut – the main interface for digestion, detoxification, and redox homeostasis. Studies of insect physiology and plant defense consistently show that plant phenolics and flavonoids can impair nutrient acquisition either directly, by interacting with digestive enzymes, or indirectly, by increasing oxidative stress and forcing insects to upregulate detoxification systems such as cytochromes P450, GSTs, and antioxidant enzymes.^29^, ^30^, ^31^ Our docking results are fully compatible with this framework, because rutin and narcissin converged on precisely these vulnerable functions – digestive proteases and detoxification‐related proteins – while their distinct contact patterns suggest partially complementary modes of disruption. From an evolutionary perspective, methylation may therefore represent a plant strategy to expand chemical space around an already active scaffold: the unmethylated flavonol preserves stronger polar interactions with some protein sites, whereas the methylated derivative gains hydrophobic packing and potentially greater persistence. Such diversification would increase the probability of overcoming insect counter‐adaptation, especially in genotypes that constitutively deploy mixtures of related flavonols in tissues most exposed to herbivory.

## CONCLUSIONS

Our integrated chemical, structural, and biological analyses demonstrate that the *O*‐methylation of rutin represents a key biochemical mechanism enhancing soybean resistance to insect herbivory. LC‐MS/MS and NMR data confirmed that resistant soybean genotypes accumulate both rutin and its *O*‐methylated analogue isorhamnetin‐3‐*O*‐rutinoside (narcissin), and that methylation can be chemically reproduced through controlled synthesis with methyl iodide. Bioassays with *A. gemmatalis* revealed that methylated rutin derivatives caused a drastic reduction in larval survival – approximately 95% mortality within 5 days – whereas non‐methylated rutin reached similar lethality only after 17 days, indicating that *O*‐methylation markedly accelerates and amplifies insecticidal activity.

Molecular docking further showed that both flavonols target digestive proteases, detoxification enzymes, and gut structural proteins, with *O*‐methylation shifting ligand interactions toward hydrophobic sub‐pockets and potentially enhancing persistence and penetration in the insect midgut. These complementary chemical and biological effects support a dual‐ligand defense model, in which rutin and narcissin act synergistically to disrupt digestion and detoxification pathways. Altogether, our findings link metabolic *O*‐methylation of flavonols to a more potent anti‐herbivore phenotype and identify narcissin as a promising target for breeding and metabolic engineering aimed at improving soybean pest resistance.

Finally, the tissue‐specific accumulation of rutin and narcissin in leaves, flowers, and pods of resistant genotypes demonstrates that the flavonol *O*‐methylation pathway operates systemically, reinforcing its role as a constitutive defense mechanism concentrated in tissues most exposed to insect attack.

## FUNDING INFORMATION

This study was supported by the National Institute of Science and Technology in Plant–Pest Interaction (INCT‐IPP), Fundação de Amparo à Pesquisa de Minas Gerais (FAPEMIG), Coordenação de Aperfeiçoamento de Pessoal de Nível Superior (CAPES), and Conselho Nacional de Desenvolvimento Científico e Tecnológico (CNPq); grant numbers: FAPEMIG: APQ‐05406‐24; APQ‐03986‐24; APQ‐01338‐23. CNPq‐403 431/2025‐5.

## Supporting information


**Figure S1.** Preparative liquid chromatographic separation of enriched aqueous extract for flavonoids. Subfractions at 36–37, 38.5, 39.5, and 40.5 min were collected for LC‐MS QqQ analyses.

**Figure S2.** ^1^H‐NMR spectrum (400 MHz, CD_3_OD) of the 40.5 min retention time subfraction.

**Figure S3.** Pupal weight of *Anticarsia gemmatalis* larvae after 20 days of development under control treatments. Mean pupal weight (g) of larvae reared on artificial diets corresponding to the negative control 1 (diet + H_2_O) and negative control 2 (diet + reaction medium without rutin). No significant differences were detected between treatments, demonstrating that the reaction medium and any residual reagents from the chemical synthesis did not affect larval survival or pupal biomass. These results confirm that the reagents used in the methylation reaction alone did not exert toxic or developmental effects on *A. gemmatalis*.

**Table S1.** Residue‐level contact analysis between protease models and the flavonoids rutin and isorhamnetin‐3‐rutinoside (narcissin) obtained from molecular docking. For each residue, distances (Å) and number of contacts were calculated, with classification into shared or ligand‐specific interactions. Residues are identified by amino acid type, chain, and position. The column key provides a unique identifier for each residue, combining amino acid type, chain, and position. The columns resn_r, chain_r, and resi_r specify the amino acid, chain, and position of residues interacting with rutin, while d_rutin indicates the minimum distance (Å) and n_contacts_rutin the number of contacts with rutin. Similarly, resn_n, chain_n, and resi_n denote residues interacting with narcissin (isorhamnetin‐3‐rutinoside), with d_narc and n_contacts_narc representing the minimum distance and number of contacts, respectively. The columns resn, chain, and resi provide complementary residue identification. The column class categorizes the interaction as ‘shared’ (contact with both ligands), ‘rutin_only’, or ‘narc_only’. The column delta_d reports the difference between contact distances for the two ligands, and closer indicates which ligand is in closer proximity. Finally, res_label provides a simplified residue label (amino acid plus position).

**Table S2.** Residue‐level contact analysis between cytochrome P450 models and the flavonoids rutin and isorhamnetin‐3‐rutinoside (narcissin) obtained from molecular docking. For each residue, distances (Å) and number of contacts were calculated, with classification into shared or ligand‐specific interactions. Residues are identified by amino acid type, chain, and position. Residue‐level contact analysis between protease models and the flavonoids rutin and isorhamnetin‐3‐rutinoside (narcissin) obtained from molecular docking. For each residue, distances (Å) and number of contacts were calculated, with classification into shared or ligand‐specific interactions. Residues are identified by amino acid type, chain, and position. The column key provides a unique identifier for each residue, combining amino acid type, chain, and position. The columns resn_r, chain_r, and resi_r specify the amino acid, chain, and position of residues interacting with rutin, while d_rutin indicates the minimum distance (Å) and n_contacts_rutin the number of contacts with rutin. Similarly, resn_n, chain_n, and resi_n denote residues interacting with narcissin (isorhamnetin‐3‐rutinoside), with d_narc and n_contacts_narc representing the minimum distance and number of contacts, respectively. The columns resn, chain, and resi provide complementary residue identification. The column class categorizes the interaction as ‘shared’ (contact with both ligands), ‘rutin_only’, or ‘narc_only’. The column delta_d reports the difference between contact distances for the two ligands, and closer indicates which ligand is in closer proximity. Finally, res_label provides a simplified residue label (amino acid plus position).

**Table S3.** Kaplan–Meier survival analysis and LT50 estimates of *Anticarsia gemmatalis* larvae under different treatments.

## Data Availability

Data sharing not applicable to this article as no datasets were generated or analysed during the current study.
