# Supplementary material for: Insect‐resistant soybean genotypes accumulate rutin and its O‐methylated derivative narcissin, a more potent flavonol targeting Anticarsia gemmatalis digestive and detoxification enzymes and reducing larval survival
Source: J Sci Food Agric. 2026 Apr 18;106(10):5921–33. doi: 10.1002/jsfa.70640 (PMC13258211; doi:10.1002/jsfa.70640)
Supplement: Supplementary file 6 — Table S3. Kaplan–Meier survival analysis and LT50 estimates of Anticarsia gemmatalis larvae under different treatments. [file JSFA-106-5921-s006.docx]

| **Kaplan-Meier survival** | | | | | | | |
| --- | --- | --- | --- | --- | --- | --- | --- |
|  |  |  |  |  |  |  |  |
|  |  |  |  |  |  |  |  |
| **Treatments** | **n** | **Deaths** | **Censored** | **Mean time (days)** | **Median survival**  **(days)** | **Final survival** |  |
| Water control | 32 | 1 | 31 | 19.53125 |  | 0.96875 |  |
| Reaction medium control | 32 | 0 | 32 | 20 |  | 1 |  |
| Rutin control | 32 | 32 | 0 | 10.5 | 12 | 0 |  |
| Methylation product treatment | 32 | 32 | 0 | 4.59375 | 4 | 0 |  |
| **Global log-rank test** | | | |  |  |  |  |
|  |  |  |  |  |  |  |  |
| **Comparison** | **Chi-square** | **gl** | **p** |  |  |  |  |
| All Groups | 190.669 | 3 | 4.37 1E-41 |  |  |  |  |
|  |  |  |  |  |  |  |  |
|  |  |  |  |  |  |  |  |
|  |  |  |  |  |  |  |  |
| **Cox Model (hazard ratio)** | | | | | |  |  |
|  |  |  |  |  |  |  |  |
| **Comparison** | **Hazard ratio** | **IC95% Lower** | **IC95% Upperr** | **p** | **Notes** |  |  |
| Methylation vs Rutin control | 6.595 | 3.330 | 13.062 | 6.3E-08 | Estimable |  |  |

**Table S3:** Kaplan–Meier survival analysis and LT50 estimates of *Anticarsia gemmatalis* larvae under different treatments.
